# Supplementary figures and images for: Cryo-electron tomographic investigation of native hippocampal glutamatergic synapses
Source: eLife. 2024 Nov 4;13:RP98458. doi: 10.7554/eLife.98458 (PMC11534335; doi:10.7554/eLife.98458)

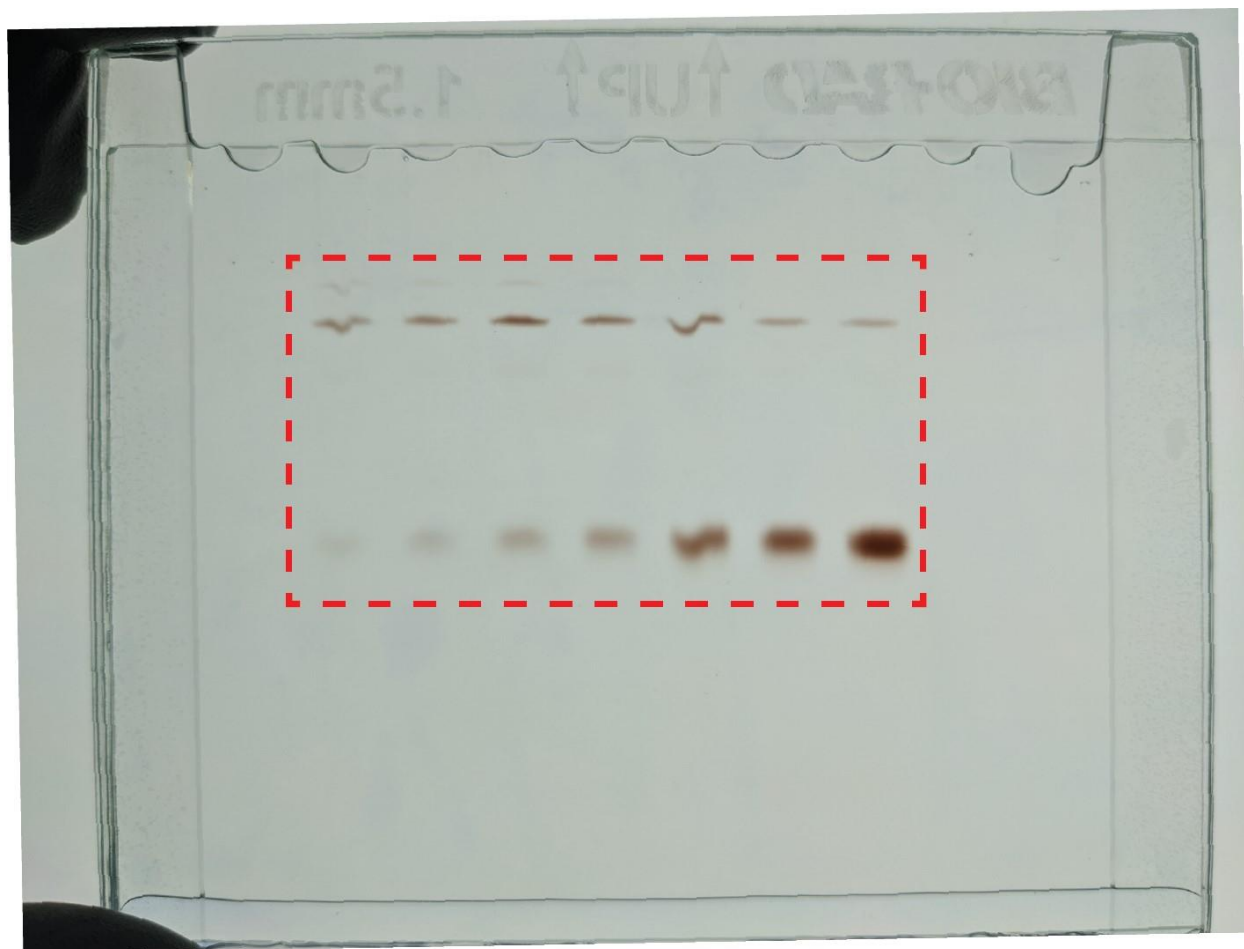

Supplement: Figure 1—source data 1. [file elife-98458-fig1-data1.pdf]

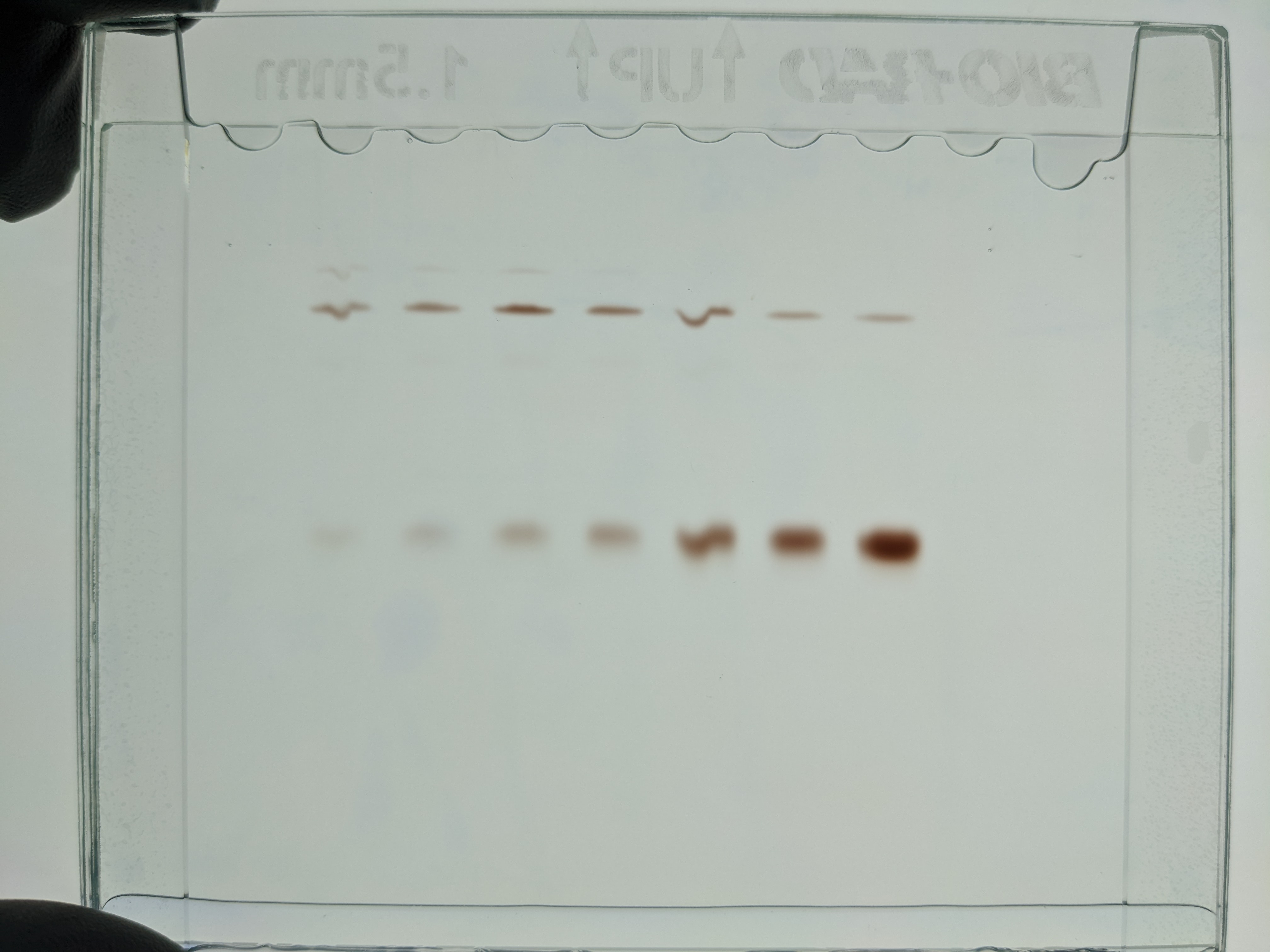

Supplement: Figure 1—source data 2. [file elife-98458-fig1-data2.zip › Figure 1-source data 2.jpg]

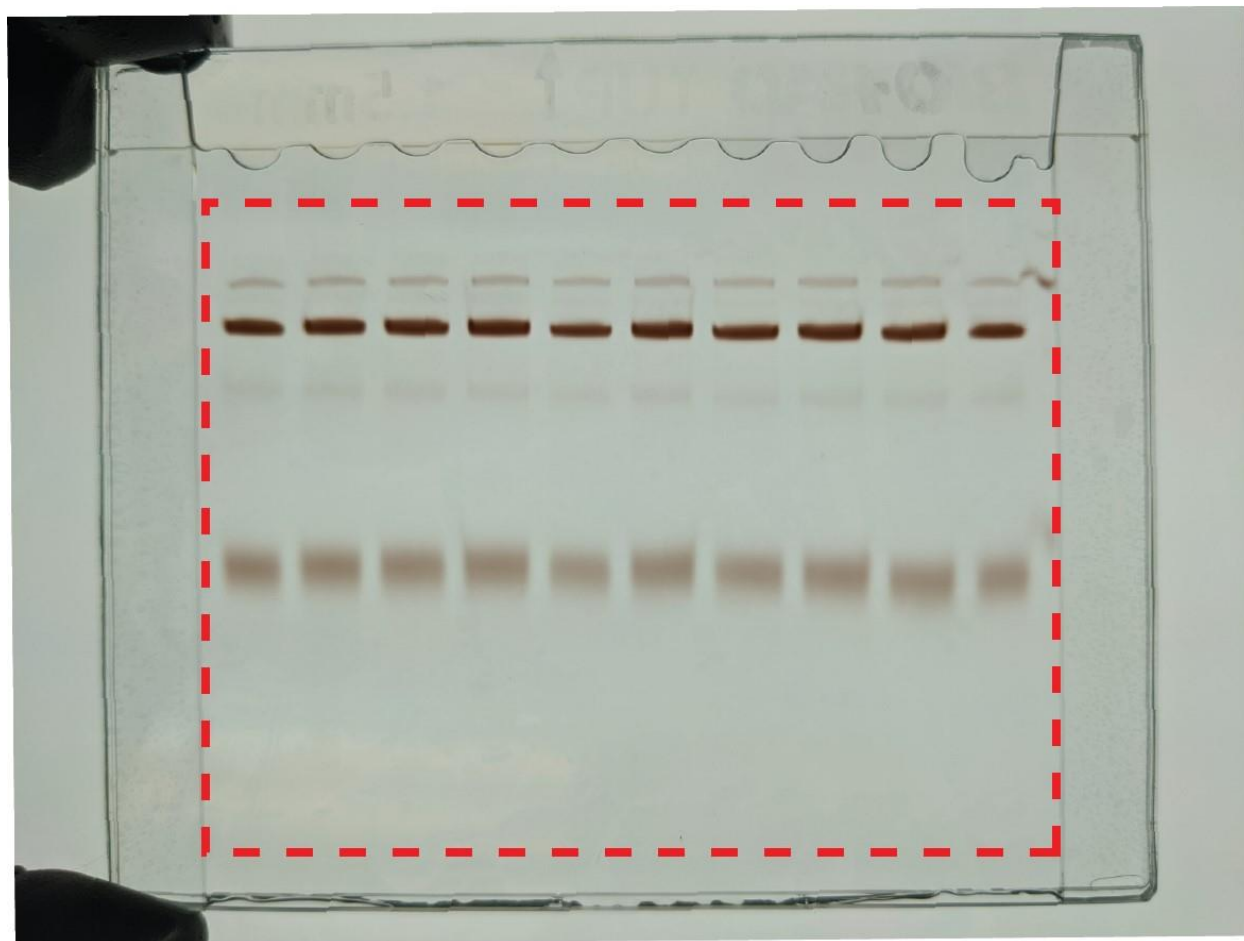

Supplement: Figure 1—figure supplement 1—source data 1. [file elife-98458-fig1-figsupp1-data1.pdf]

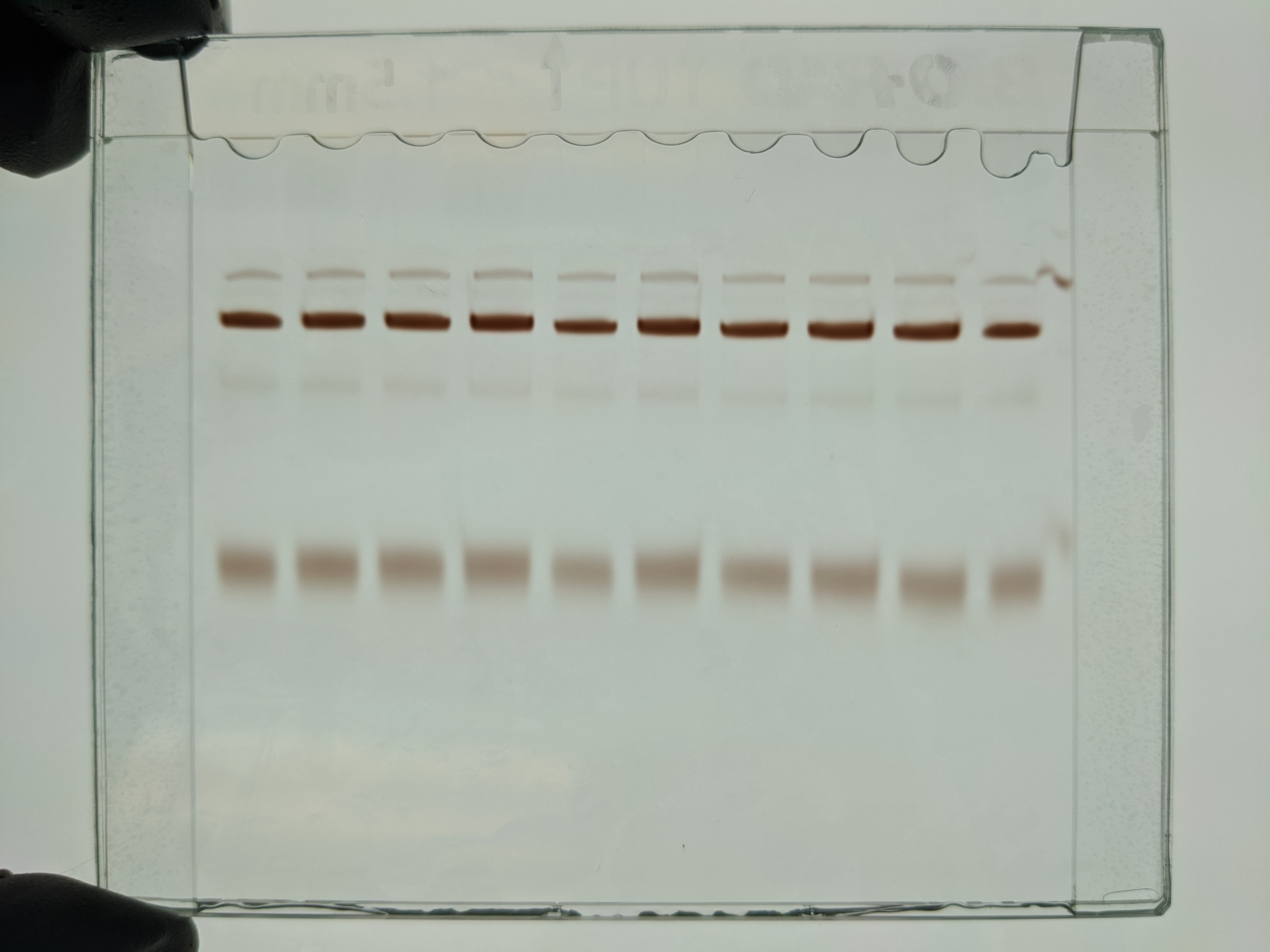

Supplement: Figure 1—figure supplement 1—source data 2. [file elife-98458-fig1-figsupp1-data2.zip › Figure 1-figure supplement 1-source data 2.jpg]
